# Outbreak of Puumala Virus Infection, Sweden

**DOI:** 10.3201/eid1405.071124

**Published:** 2008-05

**Authors:** Lisa Pettersson, Jens Boman, Per Juto, Magnus Evander, Clas Ahlm

**Affiliations:** * Umeå University, Umeå, Sweden; † County Council of Västerbotten, Umeå, Sweden

**Keywords:** Hantavirus, hemorrhagic fever with renal syndrome, outbreak, Sweden, Puumala virus, climate, snow, weather, vole, dispatch

## Abstract

An unexpected and large outbreak of Puumala virus infection in Sweden resulted in 313 nephropathia epidemica patients/100,000 persons in Västerbotten County during 2007. An increase in the rodent population, milder weather, and less snow cover probably contributed to the outbreak.

Members of the genus *Hantavirus* (family *Bunyaviridae*) are rodent-borne pathogens, and virus is transmitted to humans by inhalation of infected rodent excreta (*1*). In Sweden, Finland, Norway, Russia, and parts of central Europe, Puumala virus (PUUV) is endemic in bank voles (*Myodes glareolus*). PUUV infection in humans cause nephropathia epidemica (NE), a mild form of hemorrhagic fever with renal syndrome (HFRS). In Sweden, ≈90% of all NE cases are found in the 4 northernmost counties. Västerbotten County (Figure 1) has the highest incidence of human hantavirus infection in Sweden and probably one of the highest worldwide. Historically, the incidence rate is 20 per 100,000 persons per year (*2*), but the true incidence is considered to be 7–8 times higher (*3*).

**Figure 1:**
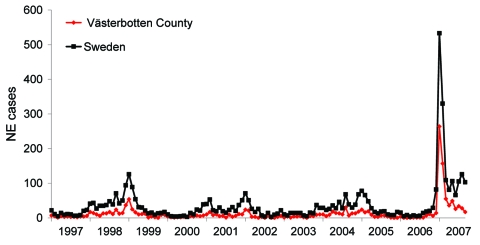
Monthly incidence of nephropathia epidemica (NE) in Sweden and Västerbotten County, Sweden, January 1997–September 2007.

There is a 3–4-year periodicity in the number of NE cases that is linked to the bank vole population dynamics in northern Sweden (*2*). After inhaling infectious aerosols originating from rodent saliva, urine, or feces, the patient has a 1–5-week incubation period before onset of disease symptoms. The most common NE symptoms are fever, headache, nausea, abdominal and back pain, vomiting, myalgia, and visual disturbance. One third of the patients have mostly mild hemorrhagic manifestations (*4*,*5*). Renal failure is typical with initial oliguria during the acute phase and polyuria in the convalescence phase. Dialysis is sometimes needed and <0.5% of NE cases are fatal. There is no effective treatment or available vaccine.

## The Study

The local University Hospital of Umeå is the reference center for diagnosis of NE serving the 4 northernmost counties of Sweden, and many patients with NE are hospitalized here. In 2007, a sudden and large outbreak of hantavirus infections occurred in northern Sweden. The outbreak peaked in January 2007 (Figure 1) with many NE patients who had a considerable effect on public health services. The NE outbreak continued in the following months, but with fewer cases than in early 2007 (Figure 1).

For NE diagnosis, we used an immunofluorescence assay to detect PUUV-reactive immunoglobulin (Ig) M and IgG antibodies in serum of all patients with clinically suspected NE (*6*). A real-time reverse transcription–PCR (*6*) was used to obtain an amplification product from 1 patient sample. This product was sequenced and the S-segment sequence obtained (GenBank accession no. EU177630) was highly homologous to those of other rodent PUUV isolates from the area.

NE is a reportable disease under the Swedish Communicable Diseases Act. The outbreak peaked during the first 3 months of 2007; 972 cases were recorded in Sweden and 474 cases in Västerbotten County. NE patients mostly showed classic HFRS symptoms and mild to severe disease requiring hospitalization and occasionally intensive care. Accordingly, as many as 30% of the patients whose conditions had been diagnosed as NE were hospitalized, and 2 known deaths (case-fatality rate 0.25%) in the 2 northernmost counties in Sweden were recorded during the first 3 months of 2007. No patient had to continue dialysis after the acute phase of the disease.

We detected PUUV RNA in the milk of 2 breastfeeding women with a diagnosis of NE. Their children did not show any clinical symptoms of NE. However, we did not have access to samples to analyze whether the children had asymptomatic infections. Three pregnant women also had received a diagnosis of NE, but no clinical evidence of transmission from mother to child was reported. Analyses of the placentas did not detect any PUUV RNA. Only maternal IgG antibodies to PUUV were found in blood from umbilical cords. One woman miscarried after 12 weeks of pregnancy 3 weeks before showing symptoms of clinical NE, and death of the fetus may have been caused by viremia during the incubation period. During the peak of the outbreak (December 2006–March 2007), 488 cases occurred in Västerbotten County, and, as expected, more men (58%, 281/488) than women (42%, 207/488) had NE; most cases (72%) were among persons 35–74 years of age (Table).

**Table:** Nephropathia epidemica cases in Västerbotten County,  Sweden, December 1, 2006–March 31, 2007

Age group, y	No. (%) cases
<1–4	1 (0.20)
5–14	16 (3.3)
15–24	34 (7.0)
25–34	48 (9.8)
35–44	82 (17)
45–54	89 (18)
55–64	103 (21)
65–74	78 (16)
75–84	32 (6.6)
85–94	5 (1.0)
Total	488 (100)

The incidence of NE in Västerbotten County was 313 diagnosed cases/100,000 persons in 2007 compared with 73/100,000 in 1999, 38/100,000 in 2002, and 61/100,000 in 2005 (Figure 1). The number of NE cases usually depends on the size of the vole population, which peaks every third to fourth year (*2*,*7*). An increase in the bank vole population was reported in northern Sweden in the fall of 2006, with a trap index of 7.64. This index is similar to those of 2 NE peaks in the fall of 1998–1999 and 2004–2005 when trap indices were ≈8 (*8*). Trapping indices represents the number of voles captured per 100 trapping nights, a reflection of the relative population size on each sampling occasion (*9*). Thus, the bank vole population was high, but not more than in previous peak years and could not explain the high number of NE cases in 2007.

**Figure 2:**
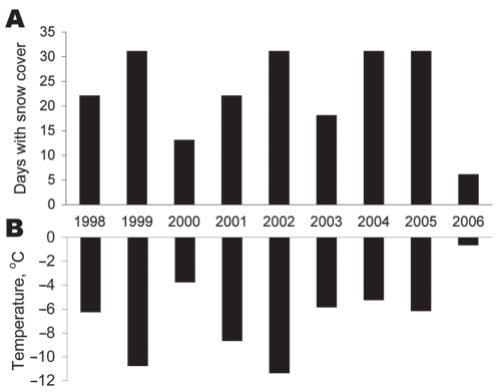
Climate conditions, December 1998–2006, in the nephropathia epidemica outbreak area of Västerbotten County, Sweden. A) Number of days with a snow cover. B) Average temperature. Snow cover was defined as a snow depth >0 cm. Measurements were made in locations ≈30 km from the coast. Data were obtained from the Swedish Meteorological and Hydrological Institute.

We considered other possible factors influencing hantavirus transmission to humans. One factor is increased exposure of humans to infected rodent excreta. We had received several reports from inhabitants in areas where bank voles normally live that more bank voles were found in traps inside houses than usual. When we investigated the weather conditions during this period, December 2006 was exceptional with respect to the mild weather with no or little snow and hard ice cover in the coastal area of northern Sweden. In Västerbotten County, the average temperature in December was 6.0°C–9.0°C warmer than normal (normally the average temperature in Västerbotten County varies by –4°C along the coast and –13°C in the mountains) The average temperature in Sweden was 4.5°C–9.5°C warmer than normal in December 2006 (Figure 2). The snow cover during winter is important for bank vole survival because bank voles have access to food below the snow and hide from predators and the cold (*10*). During 2 previous NE peak periods (2001–2002 and 2004–2005), the ground was already covered with snow in early winter (Figure 2). For these reasons, during December 2006, when the ground had no snow cover for 25 of 31 days (Figure 2), bank voles may have sought refuge in barns and houses and other buildings, thereby increasing the exposure for the human population at risk. A concurrent epizootic may have occurred among bank voles, which resulted in larger numbers of infectious animals, as shown in previous rodent studies (*11*,*12*). However, we did not have access to rodents during this period and this hypothesis needs to be studied.

## Conclusions

This report shows how a zoonotic disease can suddenly result in an unexpected and large human outbreak. Presently, the numbers of NE cases in northern Sweden are still unusually high. Data indicate that the bank vole population during the fall of 2007 increased to an even higher level and a new outbreak is forecasted (*8*). However, the size of the rodent population is not the only factor that determines the size of a hantavirus epidemic. As shown in this report, climate factors may have contributed to the recent large outbreak in northern Sweden.

## References

[1] Schmaljohn C, Hjelle B. Hantaviruses: a global disease problem. Emerg Infect Dis. 1997;3:95–104. 9204290 10.3201/eid0302.970202 PMC2627612

[2] Olsson GE, Dalerum F, Hörnfeldt B, Elgh F, Palo TR, Juto P, Human hantavirus infections, Sweden. Emerg Infect Dis. 2003;9:1395–401. 14718081 10.3201/eid0911.030275 PMC3035548

[3] Ahlm C, Linderholm M, Juto P, Stegmayr B, Settergren B. Prevalence of serum IgG antibodies to Puumala virus (haemorrhagic fever with renal syndrome) in northern Sweden. Epidemiol Infect. 1994;113:129–36. 7914872 10.1017/s0950268800051542 PMC2271219

[4] Settergren B, Juto P, Trollfors B, Wadell G, Norrby SR. Clinical characteristics of nephropathia epidemica in Sweden: prospective study of 74 cases. Rev Infect Dis. 1989;11:921–7. 2574903 10.1093/clinids/11.6.921

[5] Mustonen J, Brummer-Korvenkontio M, Hedman K, Pasternack A, Pietilä K, Vaheri A. Nephropathia epidemica in Finland: a retrospective study of 126 cases. Scand J Infect Dis. 1994;26:7–13. 10.3109/00365549409008583 7910705

[6] Evander M, Eriksson I, Pettersson L, Juto P, Ahlm C, Olsson GE, Puumala hantavirus viremia diagnosed by real-time reverse transcriptase PCR using samples from patients with hemorrhagic fever and renal syndrome. J Clin Microbiol. 2007;45:2491–7. 10.1128/JCM.01902-06 17537944 PMC1951205

[7] Brummer-Korvenkontio M, Vapalahti O, Henttonen H, Koskela P, Kuusisto P, Vaheri A. Epidemiological study of nephropathia epidemica in Finland 1989–96. Scand J Infect Dis. 1999;31:427–35. 10.1080/00365549950163941 10576121

[8] Olsson GE, Hörnfeldt B, Hjertkvist M, Lundkvist Å. Nephropathia epidemica: high risk in Norrland during winter [in Swedish]. Lakartidningen. 2007;104:3450–3. 18072613

[9] Olsson GE, White N, Ahlm C, Elgh F, Verlemyr AC, Juto P, Demographic factors associated with hantavirus infection in bank voles (*Clethrionomys glareolus*). Emerg Infect Dis. 2002;8:924–9. 12194768 10.3201/eid0809.020037 PMC2732544

[10] Hansson L, Hentonnen H. Gradients in density variations of small rodents: the importance of latitude and snow cover. Oecologia. 1985;67:394–402. 10.1007/BF00384946 28311574

[11] Ahlm C, Alexeyev OA, Elgh F, Aava B, Wadell G, Tärnvik A, High prevalence of hantavirus antibodies in bank voles (*Clethrionomys glareolus*) captured in the vicinity of households afflicted with nephropathia epidemica. Am J Trop Med Hyg. 1997;56:674–8. 9230802 10.4269/ajtmh.1997.56.674

[12] Escutenaire S, Chalon P, Verhagen R, Heyman P, Thomas I, Karelle-Bui L, Spatial and temporal dynamics of Puumala hantavirus infection in red bank vole (*Clethrionomys glareolus*) populations in Belgium. Virus Res. 2000;67:91–107. 10.1016/S0168-1702(00)00136-2 10773322

